# Recovery of stem cell proliferation by low intensity vibration under simulated microgravity requires LINC complex

**DOI:** 10.1038/s41526-019-0072-5

**Published:** 2019-05-15

**Authors:** H. Touchstone, R. Bryd, S. Loisate, M. Thompson, S. Kim, K. Puranam, A. N. Senthilnathan, X. Pu, R. Beard, J. Rubin, J. Alwood, J. T. Oxford, G. Uzer

**Affiliations:** 1 Department of Mechanical and Biomedical Engineering, Boise State University, Boise, ID 83725 USA; 2 Department of Medicine, University of North Carolina Chapel Hill, Chapel Hill, NC 27599 USA; 3 Biomolecular Research Center, Boise State University, Boise, ID 83725 USA; 4 Space Biosciences Division, NASA-Ames Research Center, Mountain View, CA 94035 USA

**Keywords:** Stem cells, Biomedical engineering

## Abstract

Mesenchymal stem cells (MSC) rely on their ability to integrate physical and spatial signals at load bearing sites to replace and renew musculoskeletal tissues. Designed to mimic unloading experienced during spaceflight, preclinical unloading and simulated microgravity models show that alteration of gravitational loading limits proliferative activity of stem cells. Emerging evidence indicates that this loss of proliferation may be linked to loss of cellular cytoskeleton and contractility. Low intensity vibration (LIV) is an exercise mimetic that promotes proliferation and differentiation of MSCs by enhancing cell structure. Here, we asked whether application of LIV could restore the reduced proliferative capacity seen in MSCs that are subjected to simulated microgravity. We found that simulated microgravity (sMG) decreased cell proliferation and simultaneously compromised cell structure. These changes included increased nuclear height, disorganized apical F-actin structure, reduced expression, and protein levels of nuclear lamina elements LaminA/C LaminB1 as well as linker of nucleoskeleton and cytoskeleton (LINC) complex elements Sun-2 and Nesprin-2. Application of LIV restored cell proliferation and nuclear proteins LaminA/C and Sun-2. An intact LINC function was required for LIV effect; disabling LINC functionality via co-depletion of Sun-1, and Sun-2 prevented rescue of cell proliferation by LIV. Our findings show that sMG alters nuclear structure and leads to decreased cell proliferation, but does not diminish LINC complex mediated mechanosensitivity, suggesting LIV as a potential candidate to combat sMG-induced proliferation loss.

## Introduction

In mechanically sensitive tissues like bone, reduced mechanical challenge encountered during microgravity and bedrest contributes to a phenotype reminiscent of sedentary subjects,^1,2^ with reduced bone quality and increased fracture risk.^3^ To alleviate the detrimental effects of weightlessness, astronauts adhere to long and rigorous exercise regimens that include running and resistance training^4^ designed to reestablish mechanical challenges lost during spaceflight. Despite these rigorous exercise regimes, data from near orbit space missions show that astronauts lose an average of 1% of their bone density per month in space.^5,6^ Bone loss due to unloading is achieved by alterations in the actions of the differentiated cells present in the tissue (e.g., osteoblasts, osteocytes, adipocytes),^7^ as well as their common progenitor, the mesenchymal stem cell (MSC).^8–10^ When loading is absent, mesenchymal stem cells that renew and regenerate bone-making osteoblast populations contribute to the loss of bone quality by decreasing the output of available osteoblasts.^2^ For example, hind limb unloading models that simulate weightlessness cause decreased proliferative and osteoblastic capacity in MSCs.^2^ Correspondingly, in unloaded humans, bone quality decreases and the bone marrow space fills with adipocytes derived from local marrow MSC.^11,12^

To maintain bone quality, MSCs rely on mechanical signals from their environment to control gene expression required for cell growth and differentiation. Vital for transducing regulatory mechanical information into the cell nuclei, the Linker of Nucleoskeleton and Cytoskeleton (LINC) complex^13^ not only provides mechanical coupling between the nucleus and the cytoplasmic cytoskeleton but also plays a role in providing nuclear access to molecular transducers of mechanical information such as βcatenin^14^ and YAP/TAZ,^15^ molecules involved in cell growth and differentiation. Containing multiple Nesprin1 & 2 and Sun1 & 2 protein components that connect cytoskeleton to intra-nuclear LaminA/C network,^16,17^ the LINC complex participates in regulating nuclear architecture.^18^ We and others have shown that LINC-mediated connectivity regulates actin polymerization^19,20^ as well as intermediate filament assembly within the nucleus.^21^ LINC-mediated forces, further alters nuclear pore size,^22^ chromatin dynamics,^23^ and gene expression,^21^ all of which influence MSC function.

Aimed at understanding the role of microgravity on MSC structure and function, recent research on simulated microgravity (sMG) models show that altered gravity conditions under sMG decrease structural integrity and contractility of cells.^24–26^ While much of this dysfunction is due to sMG-induced changes in cytoskeletal networks,^24–28^ possible effects of sMG to alter the structure of the cell nucleus and LINC complex are unknown. Our group has reported that LINC elements show functional similarities to cytoskeletal networks and are responsive to mechanical challenge. For example, stiffening of actin cytoskeleton in response to mechanical loading results in increased transcriptional expression of LINC elements and their nuclear anchor LaminA/C.^29^

Mechanical loading, as occurs during exercise and daily activities, is critical for maintaining musculoskeletal health.^30^ Interestingly, during daily functional activities, the mechanical environment of bone subjects resident cells to very few high-strain (2000–3000 µstrain), low-frequency (1–3 Hz) events. At the same time bone cells experience continual bombardment of low-strain (<5 µstrain), high-frequency signals that are the result of postural muscle contractions.^31^ When a muscle dynamically oscillates without any electrical stimulation, its natural frequency is between 10–50 Hz^32^ while dynamic oscillations up to 400 Hz is required to achieve maximal contraction via an external signal.^33^ These low-magnitude mechanical events in the skeleton decrease with age-related muscle weakness or disuse,^34^ providing a connection between muscle deterioration and bone loss. Application of low-magnitude mechanical signals to the musculoskeletal system, by using low intensity vibration (LIV) platforms, provides an exercise surrogate, delivering the anabolic mechanical input to preserve musculoskeletal competence. While we will refer delivery of these signals as LIV, other terms such as low-magnitude high-frequency (LMHF)^35,36^ vibrations, low-magnitude mechanical signals (LMMS),^37–40^ or whole-body vibrations (WBV)^41–43^ are also commonly used. In clinical studies, LIV is usually applied between 10 and 100 Hz. In humans, LIV has been shown to promote bone quantity and quality in women with osteoporosis (20–50 Hz),^44^ children with disabling conditions, including cerebral palsy (20–100 Hz),^45^ young women with osteoporosis (30 Hz),^46^ and augments bone accretion in child cancer survivors (30 Hz)^47^ and Crohn’s disease (30 Hz).^48^ Animal studies demonstrate that replacing the regulatory physical signals that decay with age or disuse with exogenously delivered mechanical stimulation is sufficient to increase trabecular bone density and volume,^49^ enhance bone stiffness and strength,^50^ and to slow bone loss caused by disuse.^51^ Further, LIV enhanced muscle contractility^52^ strength^53^ and cross-sectional area,^54^ showing that LIV signals are anabolic to skeletal muscle. In regards to stem cell studies, LIV is generally applied between 10 and 800 Hz.^55^ We and others have shown that LIV not only improves osteogenic and proliferative capacity,^55–57^ but slows down adipogenic differentiation.^58^ Concomitantly, depleting LINC function reduces the effectiveness of LIV to influence MSC differentiation.^59^

Therefore, in these studies we tested the hypotheses that a decrease in LINC function under microgravity contributes to the loss of MSC proliferative capacity, and that application of 0.7 g, 90 Hz LIV alleviates the effects of sMG on MSC proliferation in a LINC-dependent manner.

## Results

### Simulated microgravity (sMG) decreases cell proliferation without affecting cell viability

**Fig. 1:**
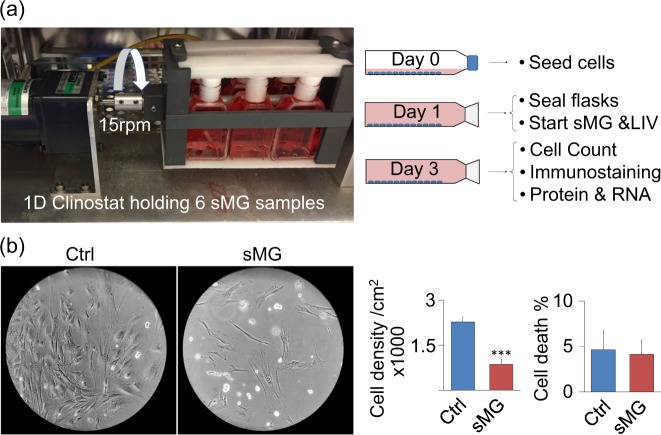
Simulated microgravity (sMG) decreases cell proliferation without affecting cell viability. **a** MSCs were subjected to simulated microgravity (sMG) via rotation at a constant 15 RPM for 3 days inside 12.5 cm^2^ sealed flasks. LIV was applied twice daily at 2 h intervals for 3 days outside the incubator at 0.7 g’s and 90 Hz for 20 min. During LIV, all samples were taken out but only sMG + LIV group was vibrated. Samples were collected for analysis 24 h after last LIV treatment. **b** Comparing the cell proliferation and dead cell to live cell ratio using microscopy indicated less cells in field of view (10 ×), flow cytometry showed that sMG decreases cell proliferation by 2.63-fold (*p* < 0.01, *n* = 6/group). Cell death was not different between difference Ctrl and sMG samples (*n* = 6/group). Group comparisons were made using unpaired T-test (**b**). *p < 0.05, **p < 0.01, ***p < 0.001, against control and each other

To test the effects of sMG on marrow derived stem cell proliferation, we subjected MSCs to a previously studied sMG regimen^60^ in the form of 15 rpm rotation on the horizontal axis. As shown in Fig. 1b, after 3 days, sMG treated group showed a 2.65-fold decrease in cell number compared with non-sMG controls (*p* < 0.01, *n* = 6/group). To query if cell death contributed to decreased cell number we first quantified the dead cells using flow cytometry. In both sMG and control groups cell death was around 5% and was not significantly different (*n* = 6/group). Next, we plated MSCs at 90–100% confluence (18,000 cell/cm^2^) and subjected to sMG for 3 days. As shown in Fig. S1, confluent, non-proliferating cultures showed no loss of cells due to sMG (*n* = 3/group).

### sMG increases nuclear height and decreases LINC elements Sun-2 and Nesprin-2G

**Fig. 2:**
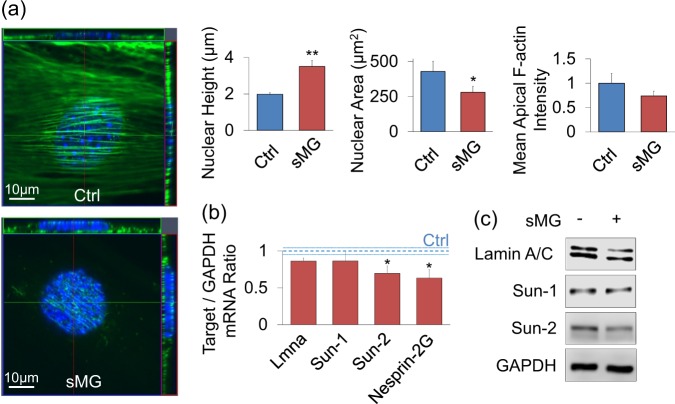
sMG increases Nuclear Height and Decrease LINC elements Sun-2 and Nesprin-2G. **a** sMG treatment reduced apical F-actin (−37%, NS) and resulted in 76% (*p* < 0.01) increase in nuclear height and 52% (*p* < 0.05) decrease in nuclear area (*n* = 15/group). Apical F-actin fibers appeared disorganized in sMG groups (see Fig. S2). **b** Quantifying the mRNA expression of nuclear structure element LaminA/C (Lmna) and LINC elements, Sun-1, Sun-2, Nesprin-1G, and Nesprin-2G showed 31% (*p* < 0.05, *n* = 9/group) and 37% (*p* < 0.05, *n* = 9/group) decrease in Sun-2 and Nesprin-2G. Lmna and Sun-1 did not show a significant change. Nesprin-1G was not detected. **c** Protein levels of laminA/C and Sun-2 in SMG treated groups were lower than controls while Sun-1 showed no difference. Group comparisons were made using Mann–Whitney U-test (Fig. 2a) and One-way ANOVA followed by a Newman–Keuls post hoc test (**b**). *p < 0.05, **p < 0.01, ***p < 0.001, against control and each other

To test if sMG caused alterations in nuclear morphology and nuclear structural proteins such as Lamin and LINC elements, we have immunostained to visualize the nuclear structure and apical F-actin organization. As shown in Fig. 2a, after 3 days of sMG, nuclear height increased by 76% in sMG groups compared to controls (*p* < 0.01, *n* = 15/group), suggesting a change in actin cytoskeleton. Correspondingly, nuclear area decreased by 52% in sMG groups (*p* < 0.01, *n* = 15/group). As shown in Fig. S2, apical F-actin in sMG groups appeared to have rearranged and the mean apical F-actin intensity was 23% lower in sMG cells, but this did not reach significance (Fig. 2a, NS, *n* = 15/group). This suggests that the changes in nuclear height may be a consequence of reduced actin connecting elements on the nuclear envelope. qPCR analysis showed that mRNA expression levels of LINC elements Nesprin-2G and Sun-2 decreased by 37% and 31% in sMG groups when compared with controls (*p* < 0.05, *n* = 9/group) while mRNA expression for Sun-1 and nuclear lamina protein LaminA/C did not change (Fig. 2b) and Nesprin-1G was not detected via qPCR. Western blot analysis showed that Sun-2 and laminA/C protein were decreased by sMG, while Sun-1 was not (Fig. 2c). Nesprin-1G (1000 kD) and Nesprin-2G (800 kD) levels were not visualized due to very large protein sizes. Together, these data indicate that sMG decreases transcription and translation of LINC elements.

### LIV rescue of sMG-induced proliferation loss requires LINC complex

**Fig. 3:**
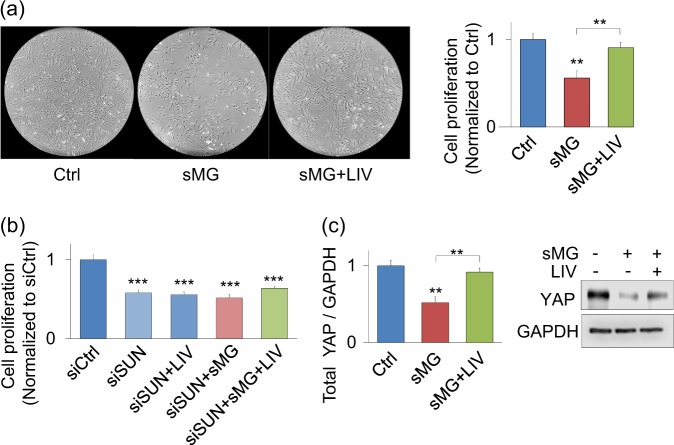
LIV rescue of sMG-induced proliferation loss requires LINC complex. **a** sMG decreased cell count by 44% after 3 days (*p* < 0.01, *n* = 8/group). LIV rescued this phenotype and increased cell count to 90% control (ns, *n* = 8/group). **b** Disabling of LINC function via siRNA against Sun-1 and Sun-2 proteins decreased the basal cell proliferation by 42% (*p* < 0.01, *n* = 8/group) addition of sMG did not decrease the cell proliferation further and LIV was unable to rescue this this decrease in both sMG and non-rotated groups. **c** After 3 days of sMG, YAP levels were decreased by 49% when compared to controls (*p* < 0.01, *n* = 3/group). Application of LIV increased the YAP levels by 40% when compared with sMG alone (*p* < 0.01, *n* = 3/group). Difference between Ctrl and sMG + LIV groups were not statistically significant. Group comparisons were made using One-way ANOVA followed by a Newman–Keuls post hoc test (**a**–**c**). *p < 0.05, **p < 0.01, ***p < 0.001, against control

We have shown that LIV increases MSC proliferation,^56^ leads to increased expression of LINC elements and results in a stiffer MSC structure.^29^ Here we intended to understand whether LIV could rescue structural losses due to sMG, thereby rescuing the proliferative defect. As shown in Fig. 3a, we used both visual inspection and cell count to compare control, sMG treated and sMG + LIV treated MSCs. sMG decreased cell proliferation by 44% (*p* < 0.01, *n* = 8/group). In sMG + LIV groups LIV was applied twice daily separated by 2 h using 0.7 g magnitude at 90 Hz frequency for 20 min. We and others have reported that separating two LIV bouts by a rest period was effective in both in vitro and in vivo models.^61,62^ Compared with sMG alone, the addition of LIV increased cell proliferation by 60% (*p* < 0.01, *n* = 8/group) restoring the cell count to 90% of the control levels.

While LINC element Sun2 and LaminA/C were decreased by sMG, LIV rescue of cell proliferation suggested that LIV mechanoresponse was intact in sMG treated cells. Therefore we tested the requirement of LINC complex in LIV rescue of cell proliferation. As we have reported previously,^14,59^ LINC complex function can be disabled by co-depleting Sun-1 and Sun-2 proteins via siRNA. LIV and sMG applied concomitantly in Sun-1 and Sun-2 depleted cells (siSUN). As shown in Fig. 3c, compared with control siRNA, siSUN decreased cell proliferation by 42% (*p* < 0.01, *n* = 8/group). In cells treated with siSUN, LIV failed to restore cell proliferation, suggesting that LIV effect on cell proliferation is dependent on a functional LINC complex. Applying 3 days sMG regimen to siSUN treated cells did not further reduce cell proliferation and LIV failed to recover cell proliferation in siSUN + sMG + LIV groups, establishing the requirement of intact LINC complex function for LIV effect.

YAP is a known regulator of cell growth and proliferation.^63^ When MSCs are grown on stiff surfaces or subjected to mechanical strain that induce adaptations in the actin cytoskeleton, YAP enters the nucleus and binds to its co-transcriptional activator TEAD to increase cell proliferation.^64^ We asked whether decreased proliferation due to sMG was due to decreased cellular YAP. Interestingly, cells exposed to sMG for 3 days showed a 49% decrease in overall YAP levels (Fig. 3c, *p* < 0.01, *n* = 3/group). Twice daily application of LIV increased cellular YAP by 40% when compared with sMG (*p* < 0.01, *n* = 3/group), to the levels not significantly different from those of control cells.

### sMG does not alter acute Akt phosphorylation in response to LIV

**Fig. 4:**
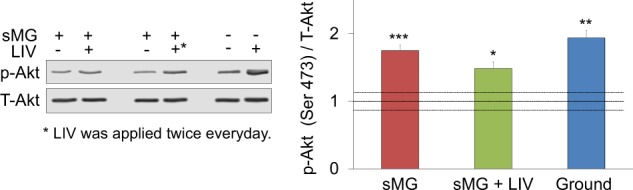
sMG does not alter acute mechanoresponse to LIV. Following 3 days of sMG, acute mechanoresponse was assessed immediately after LIV by measuring Akt phosphorylation (Ser473) while a non-LIV control served as referent. sMG group showed a 75% increase in pAkt (*p* < 0.001, *n* = 4/group). In sMG + LIV group samples were taken off the sMG device and was subjected to daily LIV regimen (2 × 20 min, 0.7 g, 90 Hz, separated by 2 h), pAkt levels were increased by 48% when compared with non-vibrated control (*p* < 0.05, *n* = 4/group). Ground control was not subjected to sMG or LIV and showed a 93% increase in pAkt following single LIV regimen (*p* < 0.01, *n* = 4/group). No difference was found between sMG, sMG + LIV, and ground treatments. Black dashed lines correspond to non-LIV controls and their combined deviation. Group comparisons were made using One-way ANOVA followed by a Newman–Keuls post hoc test. *p < 0.05, **p < 0.01, ***p < 0.001, against control and each other

Although LIV was effective in restoring proliferation in sMG treated cells, we wished to confirm that LIV signaling was present, as a full LIV mechanoresponse relies on LINC.^59^ LIV transiently phosphorylates Akt at Ser473 residue in a LINC-dependent manner.^59^ To test the effect of LIV and sMG on acute cell mechanosensitivity we used three groups. The sMG group was subjected to 3 days of sMG, and on day 4, two sets of flasks were taken out of the sMG system and were subjected to a double LIV regimen (2 × 20 min LIV at 0.7 g, 90 Hz, separated by 2 h). As shown in Fig. 4, in sMG treated cells, LIV induced a 75% increase in pAkt (*p* < 0.01, *n* = 4/group). In sMG + LIV group, each day, sMG samples were removed from the clinostat and subjected to the LIV regimen. On day 4, cell lysates were collected immediately after LIV regimen. In sMG + LIV groups, pAkt levels were increased 48% when compared with non-vibrated controls (*p* < 0.05, *n* = 4/group). Finally, non-sMG controls were not subjected to sMG or LIV (Ground). Double LIV regimen on day 4 increased p-Akt levels by 93% (*p* < 0.01, *n* = 4/group). Basal pAkt levels were not significantly different between sMG, sMG + LIV and Control groups.

### LaminA/C, Sun-2, and p-FAK levels decreased by sMG were recovered by LIV

**Fig. 5:**
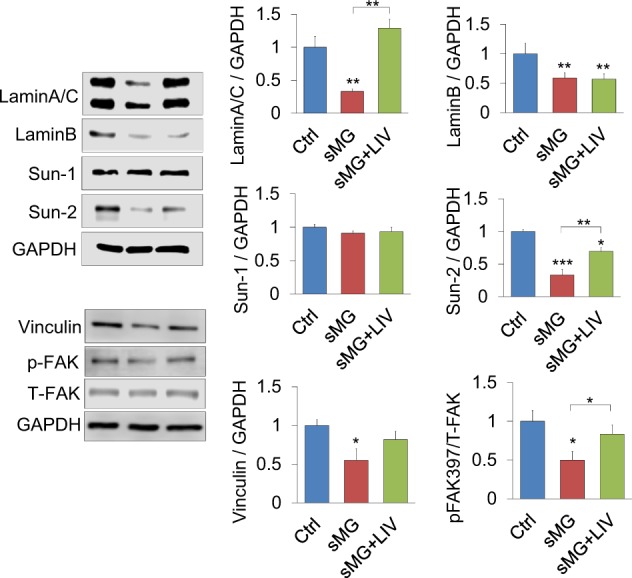
LaminA/C, Sun-2 levels, and FAK phosphorylation decreased by sMG, were recovered by LIV. We tested the effect of LIV on the structural proteins of nucleus and focal adhesion. sMG, decreased the amount of nuclear proteins LaminA/C (−67%, *p* < 0.01), LaminB (−41%, *p* < 0.01), and Sun-2 (−66%, *p* < 0.001) while Sun-1 showed no change. Both focal adhesion protein Vinculin and focal adhesion kinase phosphorylation at Tyr 397 residue (pFAK), indicative of integrin engagement, were decreased in response to sMG by 46 and 51% (*P* < 0.05). Addition of daily LIV regimen partially recovered levels of Lamin/AC (+105%, *p* < 0.01), Sun-2 (+36%, *p* < 0.01), and p-FAK (+23%, *p* < 0.05), but did not change LaminB, Sun-2, and Vinculin. *n* = 3/group for all experiments. Group comparisons were made One-way ANOVA followed by a Newman–Keuls post hoc test. *p < 0.05, **p < 0.01, ***p < 0.001, against control and each other

We tested the effect of chronic sMG and LIV on the basal levels of nuclear and focal adhesion proteins. Nuclear proteins included LaminA/C, laminB1, Sun-1, and Sun-2. Focal adhesion proteins included Vinculin and FAK. Together with total FAK levels we also probed FAK phosphorylation at Tyr 397 residue (pFAK) which was shown to be indicative of integrin engagement.^65^ As no changes in basal phosphorylated and total Akt were detected, this measure was excluded. As shown in Fig. 5, sMG decreased nuclear proteins LaminA/C by 67% (*p* < 0.01, *n* = 3/group), LaminB1 by 41% (*p* < 0.01, *n* = 3/group) and Sun-2 by 66% (*p* < 0.001, *n* = 3/group). There was no change in Sun-1 as expected (Fig. 2). Basal pFAK levels and its target Vinculin, were decreased in cells exposed to 3 days of sMG by 46% and 51%, respectively (*P* < 0.05, *n* = 3/group). Addition of daily LIV regimen during sMG partially recovered Lamin/AC (+105%, *p* < 0.01) and Sun-2 (+36%, *p* < 0.01), as well as basal p-FAK (+23%, *p* < 0.05). The decrease in LaminB1 and Vinculin due to chronic sMG was not improved by the daily LIV regimen.

### Cell-cycle proteins are dysregulated by sMG

To reveal the proteins that were regulated by sMG and LIV in MSCs we have performed a LC–MS/MS-based proteomic analysis in Ctrl, sMG, and sMG + LIV experiments. As shown in table S1, proteomic analysis found that sMG decreased 21 proteins more than two-fold, five proteins were downregulated more than four-fold and these included RanGAP1 (Ran GTPase-activating protein 1) and cell cycle regulator CDK1 (cyclin-dependent kinase 1). It has been reported that RanGAP1 plays a role in the recruitment of CDK1 into nucleus to mediate mitotic spindle assembly during cell cycle.^66,67^ Twelve proteins were upregulated more than two-fold in sMG + LIV groups compared with sMG alone. These included RanGAP1 (Ran GTPase-activating protein 1), RANG (Ran-specific GTPase-activating protein), and actin regulatory MYOC1 (Unconventional Myosin 1C). Out of 12 proteins that increased with sMG, only P4HA1 (Prolyl 4-hydroxylase subunit alpha-1), that plays a role in collagen biosynthesis,^68^ was upregulated more than four-fold. Upon LIV, ten of those proteins were downregulated under sMG were back to control levels or below in sMG + LIV groups.

## Discussion

In this study, we showed that sMG decreased cell proliferation in MSCs and reduced the cellular levels of LINC complex and lamina elements. Application of LIV restored cell proliferation and partially recovered LINC element Sun-2 and Lamina element LaminA/C. The effects of LIV to rescue cell proliferation was dependent upon LINC complex. This indicates that LIV, through effects on LINC, can be a countermeasure for sMG associated decrease in proliferation.

Our findings have shown that cell nucleus was less constrained by the cytoskeleton which resulted in an increased height, decreased area, and disorganized apical F-actin after 3 days of sMG. We and others have shown that loss of LINC complex function through dominant negative nesprin-KASH fragments, or depletion of LINC elements such as Sun or Nesprin isoforms causes similar changes in nuclear geometry.^18,69–72^ Consistent with previous studies,^73,74^ sMG also reduced the focal adhesion protein Vinculin and reduced the phosphorylated FAK levels in MSCs, suggesting reduced integrin engagement. These findings suggest that dynamic alteration of gravitational direction under simulated microgravity leads to deterioration of cytoskeletal and nucleoskeletal structures. Proteomics revealed a loss in nuclear proteins Ran-GTP and CDK1 that play a role in regulation in cell cycle and nuclear protein transport.^66,67^ In support of decreased cell proliferation our data shows that cell death was not altered by sMG, which suggested that observed changes may be due to loss of cellular structural elements.

One limitation of our studies is that sMG is not real microgravity but a clinostat-based model of microgravity. Clinostat-based microgravity models have been used in number of scientific studies to model aspects of microgravity since 70s.^75^ The basic principle of this approach is based on alteration of gravitational vector through solid body rotation around a horizontal axis.^76^ Despite this limitation, clinostat models are able to replicate the effects of real microgravity on cellular cytoskeletal organization^24,27^ which made this an ideal model for current study to look nuclear structure and LINC complex. A possible confounding factor of clinostat-based sMG and LIV studies is the fluid shear. While fluid shear stress can be significant factor in suspension cell models, horizontal axis rotation devices do not generate discernable fluid shear at flask walls to affect adherent cells.^77^ For LIV-induced fluid shear, sealing of flasks would largely eliminate fluid shear and sealing culture plates and flasks to avoid fluid motion is a commonly utilized method in both LIV^78,79^ and sMG based studies.^80^ We previously tested the possible contribution of fluid shear to LIV response.^81^ Increasing fluid shear up to 2 Pa under 100 Hz horizontal vibration did not generate a statistically significant effect when compared with LIV condition that generated 0.015 Pa fluid shear, suggesting that fluid shear does not contribute to LIV response. We have also reported the fluid shear generated by vertical 0.7 g, 90 Hz LIV vibrations in 6-well culture plates.^59^ This fluid shear is independent of fluid volume and is a result of deformations of a fluid-bounded well-bottom due to vertical accelerations.^82^ These dynamic accelerations result in lateral fluid motion over attached cells. We reported that the signal used in this study (0.7 g, 90 Hz) results in a peak velocity difference of 0.00004 m/s between fluid and well-bottom, corresponding to peak fluid shear of 0.0008 Pa in 6-well plates, well below 0.5 Pa fluid shear value that affect bone cells.^83^ In this current study we have utilized 12.5 cm^2^ culture flasks which have a 30% more surface area compared with 6-welll plates (9.5 cm^2^). While this difference in area may result in larger peak deformations and thus generate larger fluid shear, to reach 0.5 Pa, fluid shear need to increase 625-fold which is unexpected. Therefore we believe that using 12.5 flasks would not be different than 6-well culture plates in terms of LIV-induced fluid shear and thus negligible.

We recently reported that both nucleus and LINC-mediated cytoskeletal connections to nucleus are integral to maintaining the cellular tension.^84^ In this way, LINC-mediated cytoskeletal mechanical forces influence focal adhesion dynamics,^85^ perinuclear actin structures,^15^ and nuclear pore size.^22^ Inside the nucleus, LINC complex influences intra-nuclear chromatin structures^86^ as well as their interaction with inner nuclear envelope to direct processes such as DNA repair.^87^ Furthermore, disruption of LINC complexes also impedes the functionality of molecular transducers of mechanical force such as βcatenin and YAP/TAZ. For example, we have shown that disabling LINC function via co-depletion of LINC elements Sun-1 and Sun-2 impedes the nuclear entry of βcatenin by limiting its Nesprin-mediated interaction with nuclear envelope.^14^

Similarly, changes in nuclear pore size via disabling of LINC function limits YAP/TAZ nuclear entry.^22^ The forces generated at the perinuclear focal adhesion/actin assemblies require LINC complex mediated actin cap as well as LaminA/C to be present in order to exert mechanical force on nucleus and mediate force-induced YAP nuclear entry.^15^ According to our data, sMG-mediated decrease of Sun-2 and LaminA/C levels were accompanied by disorganized actin cap (Fig. S2) and reduced YAP levels after following 3 days of sMG (Fig. 3c). When LINC function was disabled via Sun-1/2 co-depletion, sMG did not further decrease proliferation. While these findings do not necessarily establish direct causality, they suggest that loss of nuclear structural elements (i.e., LINC complex and Lamin A/C) plays a role in observed decrease in cell proliferation under sMG by altering YAP functionality.

Low intensity vibration (LIV) is an exercise mimicker. In humans, LIV has been shown to promote bone quantity and quality in women with osteoporosis,^44^ and children with cerebral palsy.^45^ LIV signal frequency in this study was chosen as 90 Hz, which we reported to be efficacious on C3H10T1/2 cells,^61^ primary mouse MSCs,^59^ and MSC-derived osteocytes.^88^ While there is no consensus on which LIV signal may be more appropriate. A 2018 meta-analysis study of all LIV trials on postmenopausal women showed that signals above 20 Hz was beneficial with no statistical differences between higher frequencies.^44^ Another 2018 study performed meta-analysis of all LIV studies performed on children with cerebral palsy, which indicated beneficial effect of liv at and up to 100 Hz, including 90 Hz.^45^ While large variation in clinical trials makes comparison relatively hard to tease out the contribution of frequency to overall response, studies in rodents indicate that 90 Hz signal is generally more beneficial when compared with 45 Hz. For example, we and others reported 90 Hz to be more beneficial when compared with 45 Hz in its anabolic effect in bone,^89^ muscle,^90^ and protection against unloading-induced intervertebral disc degeneration.^91^ In studies with osteoblasts and osteocytes, we and others shown LIV efficacy between 10 and 100 Hz. While our result indicate a small positive effect of increasing LIV frequency between 10 and 100 Hz on osteoblast Cox-2 expression,^81^ we found no effect of increasing LIV frequency on gap junctional communication in osteocytes between 30 and 100 Hz.^92^ Others have reported increased LIV efficacy at 100 Hz when LIV frequency was varied between 5 and 100 Hz in osteoblasts.^78^ These studies with osteoblasts and osteocytes generally agree with rodent models that higher LIV frequencies are more efficacious compared with lower frequencies within 10–100 Hz range.

In stem cells, LIV promotes both cell proliferation and osteogenic differentiation,^56^ while slowing down adipogenesis.^59^ LIV was reported to be beneficial to MSC proliferation and/or osteogenesis between 10 and 800 Hz.^55^ In the 2T3 pre-osteoblast cell line, application of LIV was able to alleviate the decreased mineralization caused by sMG.^93^ We have further reported that LIV increases cell stiffness and expression of LINC elements.^29^ Here, we showed that twice daily application of LIV restored both proliferation and YAP levels comparable with control MSCs. LIV further increased the protein levels of LaminA/C, Sun-2, and p-FAK while laminB1 and Vinculin levels remained unchanged. This suggests that the loss of cell structure due to sMG can be in part alleviated by concomitant application of LIV.

When LINC complex function was disabled, LIV failed to rescue dysfunction induced by sMG. This suggests that LIV rescue of cell proliferation requires an intact LINC complex. As LIV mechanoresponse cannot occur when LINC is disrupted,^59^ this suggests that some of LINC function is preserved, as we confirmed here by showing that LIV was able to activate Akt in cells exposed to sMG. This suggests that sMG-induced LINC dysfunction resulted in changes in the cell-cycle mechanism, but the remaining structural elements were sufficient to support LIV signaling and consequent repair of LINC.

In summary, we have illustrated that sMG is detrimental to nuclear proteins LaminA/C, LaminB1 as well as LINC element Sun-2. These changes were accompanied by heightened nucleus, disrupted apical actin structure, reduced YAP, Vinculin, and p-FAK levels as well as decreased cell proliferation. Under these conditions MSCs remained responsive to LIV and application of daily LIV alleviated the loss of LaminA/C, Sun-2, p-FAK, and YAP levels and recovered cell proliferation but failed to rescue LaminB1 and Vinculin. These findings indicate that short bouts of daily LIV application can be effective at supporting rigorous exercise regimens designed to keep astronauts healthy. In addition, LIV-based bioreactor systems can be implemented utilized in space stations and long-term space missions to aid and augment regenerative or tissue engineering approaches.

## Materials and methods

### Cell culture

Primary mdMSCs were isolated from 8- to 10-week male C57BL/6 mice were prepared after Peister et al.^94^ Tibial and femoral marrow were collected in RPMI-1640, 9% FBS, 9% HS, 100 μg/ml pen/strep, and 12 μM L-glutamine. After 24 h, non-adherent cells were removed by washing with phosphate-buffered saline and adherent cells cultured for 4 weeks. Passage 1 cells were collected after incubation with 0.25% trypsin/1 mM EDTA × 2 min, and re-plated in a single 175-cm^2^ flask. After 1–2 weeks, passage 2 cells were re-plated at 50 cells/cm^2^ in expansion medium (Iscove modified Dulbecco’s, 9% FBS, 9% HS, antibiotics, L-glutamine). mdMSC were re-plated every 1–2 weeks for two consecutive passages up to passage 5 and tested for osteogenic and adipogenic potential, and subsequently frozen. For experimental procedures, fetal calf serum (FCS) was obtained from Atlanta Biologicals (Atlanta, GA). Culture media, trypsin-EDTA, antibiotics, and Phalloidin-Alexa-488 were from Invitrogen (Carlsbad, CA). SB415286 was purchased from Sigma Aldrich (St. Louis, MO). L. MSCs^95^ were maintained in IMDM with FBS (10%, v/v) and penicillin/streptomycin (100 μg/ml). For primary cell extractions from animals, all procedures were approved by Boise State and University of North Carolina IACUC and we complied with all relevant ethical regulations required by these institutions.

### Pharmacological reagents and antibodies

MSC were isolated from 8- to 10-week male C57BL/6 mice were prepared after Peister et al.^94^ For transiently silencing specific genes, cells were transfected with gene-specific small interfering RNA (siRNA) or control siRNA (20 nM) using PepMute Plus transfection reagent (SignaGen Labs, Rockville, MD) according to manufacturer’s instructions. MSCs were transfected using 1 µg DNA per 100,000 cells using LipoD293 transfection reagent (SignaGen Labs) according to manufacturer’s instructions. Transfections and siRNA were applied 72 h prior to sMG. A complete list of all the reagents (Table S2), antibodies (Table S3), siRNA sequences (Table S4), and primers (Table S5) along with their final concentrations are provided in the supplementary appendix.

### sMG Clinostat model

MSCs were seeded at a density of 1800 cells/cm^2^ into 12.5 cm^2^ flasks and maintained in IMDM supplemented with FCS (10%, v/v) and antibiotics. Flasks were filled and sealed to avoid any air bubbles during experiments. sMG was applied via a clinorotator which worked by rotating about a horizontal axis at 15 rpm. The non-rotated control group was placed in the same incubator. Our experimental approach is depicted in Fig. 1a.

### Cell viability

To collect dead as well as alive cells, entire medium and trypsinized cells were resuspended in 500 µL FACS buffer (PBS + 3% FBS + 0.02% NaN_3_). Gating of flow cytometer was done using a non-rotated, non-sealed calibration sample. Dead cells were stained with 4 µL/mL of propidium iodide. Dead cell percentage was determined based on 10,000 events.

### Application of LIV

Vibrations were applied at peak magnitudes of 0.7 g at 90 Hz for 20 min at room temperature. LIV was applied twice for 20 min separated by 2 h rest period. In this study we have chosen 90 Hz, 0.7 g LIV signal. This signal frequency was shown to be more effective when compared with 45 Hz in rodent models in terms of its anabolic effect in bone,^89^ muscle,^90^ and intervertebral disc.^91^ We have further shown this signal to be efficacious in C3H10T1/2 cells,^61^ primary mouse MSCs,^59^ and MSC-derived osteocytes.^88^ This regimen is considered safe for humans within 1 h of exposure time according to ISO-261 guidelines.^96^

### Fluorescence cell staining

After 72 h of sMG treatment, cells were fixed with paraformaldehyde. Nuclei and F-actin were stained per standard protocols using Hoechst 33342 (Life Technologies) and Alexa Fluor 488 conjugated Phalloidin (Life Technologies).

### Image analysis

Following sMG treatments, cells were fixed and immunostained against F-actin (Alexa Fluor 488 Phalloidin, Life Technologies) and DNA (Hoechst 33342, Life Technologies). Using Zeiss LSM 710 confocal microscope, the entire height of individual cells was imaged at intervals of 0.15 µm using the same parameters. Confocal image stacks were deconvolved usingPost Auto Quant. Further image processing and analyses were done using NIH imageJ software (https://imagej.nih.gov/ij/). Nuclear height was quantified via summation of confocal slice thickness, whereas nuclear area was measured in maximum intensity z-projections. Apical F-actin was selected by excluding in focus basal and perinuclear F-actin. Remaining apical F-actin structures on top of the nucleus were collapsed into a single image using “Maximum Intensity Projection” (Fig. S2). Apical actin intensity was reported as the mean actin intensity within nuclear area traced by Hoechst 33342.

### Real-time RT-PCR

Total RNA was isolated and purified using the RNeasy mini kit (QIAGEN) per manufacturer’s instructions. Reverse transcription was performed with 1 μg RNA in a total volume of 20 μl per reaction. 25 μL amplification reactions contained primers at 0.5 μm, deoxynucleotide triphosphates (0.2 mm each) in PCR buffer, and 0.03 U Taq polymerase along with SYBR-green (Molecular Probes, Inc., Eugene, OR) at 1:150,000. Aliquots of cDNA were diluted 5- to 5000-fold to generate relative standard curves with which sample cDNA was compared. Standards and samples were run in triplicate. PCR products from all species were normalized for the amount of GAPDH amplicons. GAPDH has been used as a referent for mesenchymal stem cells in a clinostat-based sMG system before in respect to qPCR.^60^

### Western blotting

Whole-cell lysates were prepared using an radio immunoprecipitation assay (RIPA) lysis buffer (150 mM NaCl, 50 mM Tris HCl, 1 mM EDTA, 0.24% sodium deoxycholate, 1% Igepal, pH 7.5) to protect the samples from protein degradation NaF (25 mM), Na_3_VO_4_ (2 mM), aprotinin, leupeptin, pepstatin, and phenylmethylsulfonylfluoride (PMSF) were added to the lysis buffer. Whole cell lysates (20 μg) were separated on 9% polyacrylamide gels and transferred to polyvinylidene difluoride (PVDF) membranes. Membranes were blocked with milk (5%, w/v) diluted in Tris-buffered saline containing Tween20 (TBS-T, 0.05%). Blots were then incubated overnight at 4 °C with appropriate primary antibodies. Following primary antibody incubation, blots were washed and incubated with horseradish peroxidase-conjugated secondary antibody diluted at 1:5000 (Cell Signaling) at RT for 1 h. Chemiluminescence was detected with ECL plus (Amersham Biosciences, Piscataway, NJ). At least three separate experiments were used for densitometry analyses of western blots and densitometry was performed via NIH ImageJ software. All blots derive from the same experiment and were processed in parallel. As GAPDH was previously used as a referent for mesenchymal stem cells in a clinostat-based sMG systems for western blotting,^27^ we used GAPDH for our reference protein for densitometry. A list of primary antibodies used are given in Table S3.

### Liquid chromatography–tandem mass spectrometry (LC–MS/MS)-based proteomic analysis

Whole cell lysate (20 μg of total protein) from each sample was digested with trypsin/lys C mix (Promega, Madison, WI) following the manufacturer’s instruction. Briefly, whole cell lystates were precipitated with cold acetone to remove substances that interfere downstream LC–MS/MS analysis. Resulting protein pellets were resuspended in 8 M urea solution, reduced by dithiothreitol, and alkylated by iodoacetamide. Samples were diluted to 1 M urea concentration and incubated with trypsin/lys C overnight at 30 °C. Digested samples were desalted and purified using a reverse-phase C18 spin column. Resulting peptides were chromatographically separated on a reverse-phase C18 column (10 cm × 75 µm, 3 µm, 120 Å) and analyzed on a Velos Pro Dual-Pressure Linear Ion Trap mass spectrometer (Thermo Fisher Scientific) as described previously.^97^

Protein identification and quantification were achieved by database search using Sequest HT algorithms in Proteome Discoverer 1.4 informatics software (Thermo Fisher Scientific). Swiss-Prot protein sequence database for mouse downloaded from www.unipro.org on June 15, 2018 was used in Sequest HT spectrum search. Main search parameters included: trypsin, maximum missed cleavage site of two, precursor mass tolerance of 1.5 Da, fragment mass tolerance of 0.8 Da, static modification of cysteine carbamidomethylation (+57.021 Da), and dynamic modification of methionine oxidation (+15.995 Da). A decoy database search was performed to calculate a false discovery rate (FDR). Proteins containing one or more peptides with FDR < 0.05 were considered positively identified and reported. The number of peptide spectrum matches (PSMs) for each protein was used for protein quantification. PSMs for individual proteins identified in each sample were normalized by the total PSMs of the sample and multiplied by a factor of 10,000. Only the normalized PSMs that were higher than 5 (in either group) were reported as the abundance quantifiers for individual proteins.

### Statistical analysis

Results are presented as mean ± SEM. Densitometry and other analyses were performed on at least three separate experiments. For comparisons regarding PCR or western blot data, differences between treatments within each biological replicate were assumed to follow a normal distribution due to large mean sample size (20,000 or more cells/group), thus for these comparisons, we have used two-tailed unpaired *t*-tests or one-way ANOVA. When comparing two conditions (Fig. 1b, S1) differences between groups were identified using un-paired *t*-test. When comparing more than two conditions (Figs. 2b, 3a–c, 4 and 5) we performed by one-way analysis of variance (ANOVA) followed by Newman–Keuls post hoc tests. For other comparisons with smaller sample size, including cell morphology, we assessed normality Kolmogorov–Simirnov test (*α* = 0.05). For non-normally distributed data, we have use via two-tailed Mann–Whitney U-test (Fig. 2a) as indicated in figure legends. *P*-values of <0.05 were considered significant.

### Reporting summary

Further information on research design is available in the Nature Research Reporting Summary linked to this article.

## Supplementary information

Supplementary Material

Reporting Summary

## Data Availability

The datasets generated during and/or analyzed during the current study are available from the corresponding author on reasonable request.

## Supplementary information

**Supplementary information** accompanies the paper on the *npj Microgravity* website (10.1038/s41526-019-0072-5).
