# Supplementary material for: Recovery of stem cell proliferation by low intensity vibration under simulated microgravity requires LINC complex
Source: NPJ Microgravity. 2019 May 15;5:11. doi: 10.1038/s41526-019-0072-5 (PMC6520402; doi:10.1038/s41526-019-0072-5)
Supplement: Supplementary file 1 — Supplementary Material [file 41526_2019_72_MOESM1_ESM.pdf]

Supplementary information

Recovery of stem cell proliferation by low intensity vibration under simulated microgravity requires intact LINC complex

Touchstone H[1*], Bryd R[1*], Loisate, S[1], Thompson M[1]., Kim S[2], Puranam K[2], Senthilnathan AN[2], Pu S[3], Beard R[3], Rubin J[2], Alwood J[4], Oxford JT[3], Uzer G[1†]
[1]Mechanical and Biomedical Engineering, Boise State University
[2] Department of Medicine, University of North Carolina Chapel Hill
[3] Biomolecular Research Center, Boise State University
[4] Space Biosciences Division, NASA-Ames Research Center
***Co-first authors, † Corresponding Author**

Funding support:
NASA EPSCoR NNX15AK35A (GU), NSBRI PF04304 (GU), NASA ISGC NNX15AI04H (GU), NIH P20GM109095 (GU, JO)

Figure S1. Comparison of sMG effect on proliferation between confluent and non-confluent cultures.

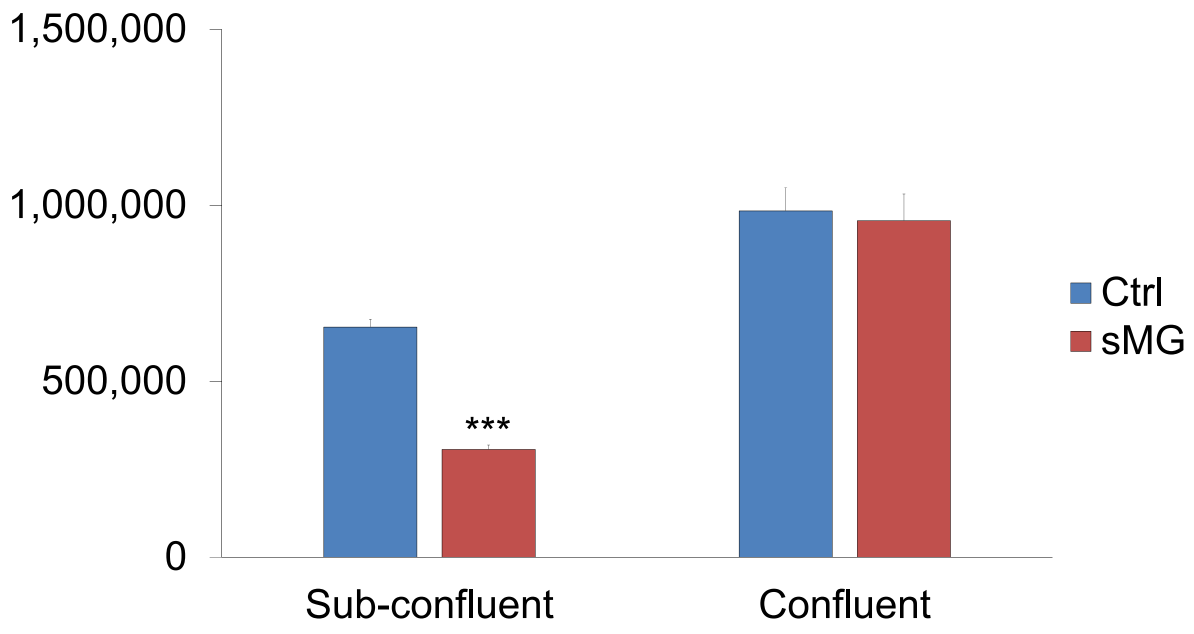

Figure S1. Comparison of sMG effect on proliferation between confluent and non-confluent cultures. 3 days of sMG decreased cell proliferation by 53% in non-confluent cultures (1,800cell/cm2, $p<0.001$, n=3/grp). There was no difference in cell proliferation when cells plated into confluent cultures (18,000 cell/cm2). Group comparisons were made using unpaired T-test (Figure 1b) * $p<0.05$, ** $p<0.01$, *** $p<0.001$, against control and each other.

Figure S2. F-actin morphology of sMG treated MSCs

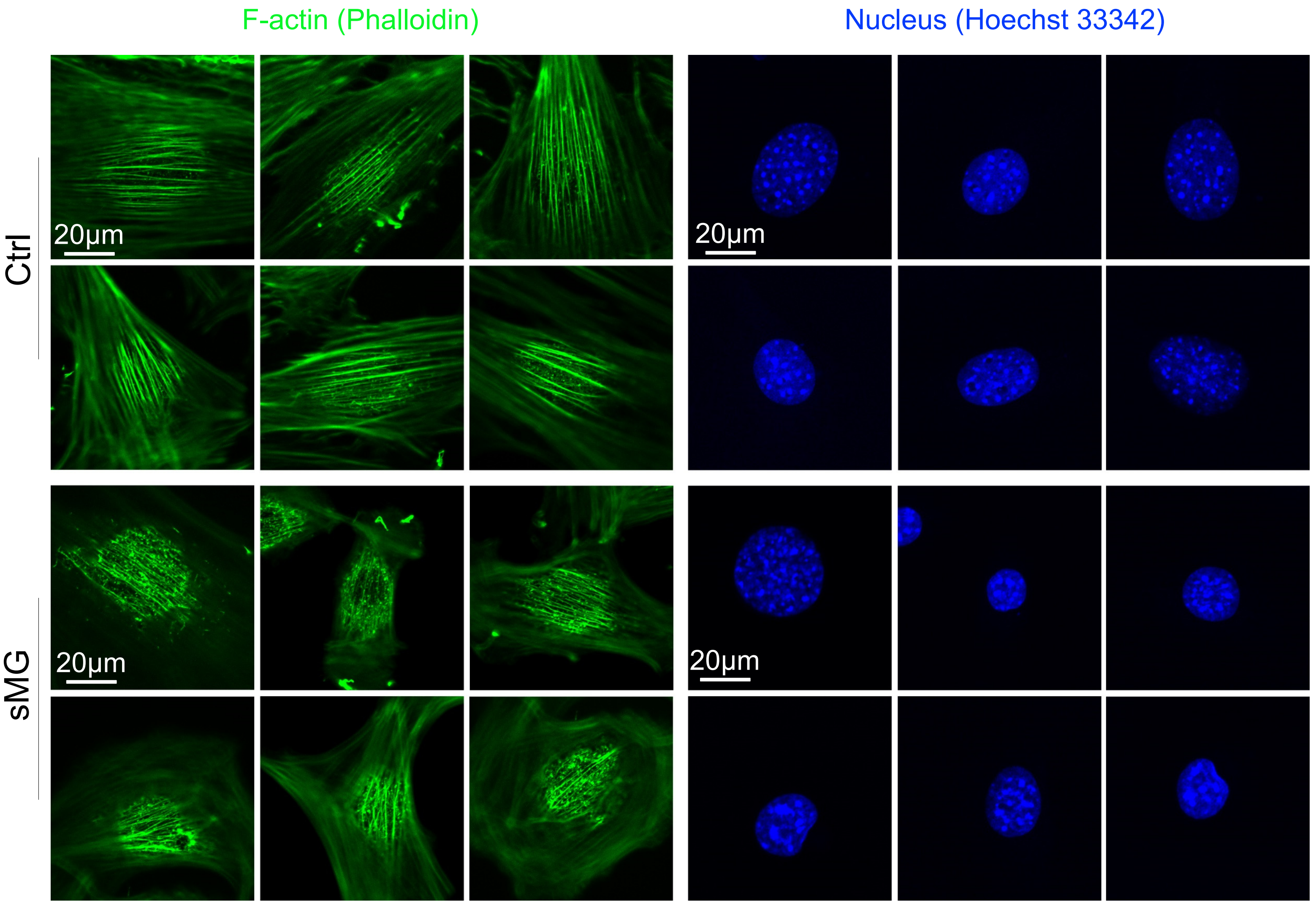

Figure S2. Representative images of actin morphology between control and sMG treated MSCs. Following sMG treatment, F-actin and nucleus were stained via Phalloidin (Green) and Hoechst 33342 (Blue) and nuclear height was measured via confocal microscopy. Apical actin structure appeared disorganized in sMG groups.

Table.S1: Differences between Control, sMG, sMG+LIV as determined by proteomics

Accession	Description	Normalized PSMs			Group Comparisons (fold of change)		
		Ctrl	sMG	sMG +LIV	sMG vs Ctrl	sMG+LIV vs sMG	sMG+LIV vs Ctrl
**P46061**	Ran GTPase-activating protein	8.79	1.75	5.17	0.20	2.95	0.59
**P11440**	Cyclin-dependent kinase 1	7.33	1.75	1.72	0.24	0.98	0.24
**Q91YQ5**	Dolichyl-diphosphooligosaccharide--protein glycosyltransferase subunit 1	7.33	1.75	8.61	0.24	4.92	1.18
**Q99LT0**	Protein dpy-30 homolog	7.33	1.75	3.44	0.24	1.97	0.47
**Q99LX0**	Protein/nucleic acid deglycase DJ-1	7.33	1.75	3.44	0.24	1.97	0.47
**O35639**	Annexin A3	5.86	1.75	6.89	0.30	3.94	1.18
**P61089**	Ubiquitin-conjugating enzyme E2 N	5.86	1.75	3.44	0.30	1.97	0.59
**Q61699**	Isoform HSP105-beta of Heat shock protein 105 kDa	5.86	1.75	3.44	0.30	1.97	0.59
**Q8R1F1**	Niban-like protein 1	5.86	1.75	6.89	0.30	3.94	1.18
**Q9CPY7**	Isoform 2 of Cytosol aminopeptidase	5.86	1.75	6.89	0.30	3.94	1.18
**P99029**	Isoform Cytoplasmic+peroxisomal of Peroxiredoxin-5, mitochondrial	10.26	3.50	8.61	0.34	2.46	0.84
**P62960**	Nuclease-sensitive element-binding protein 1	13.19	5.25	10.33	0.40	1.97	0.78
**P43275**	Histone H1.1	8.79	3.50	10.33	0.40	2.95	1.18
**Q64152**	Isoform 2 of Transcription factor BTF3	8.79	3.50	3.44	0.40	0.98	0.39
**Q8BMS1**	Trifunctional enzyme subunit alpha, mitochondrial	8.79	3.50	5.17	0.40	1.48	0.59
**Q9WTI7**	Isoform 2 of Unconventional myosin-Ic	4.40	1.75	5.17	0.40	2.95	1.18
**Q9DCL9**	Multifunctional protein ADE2 OS=Mus musculus	11.73	5.25	3.44	0.45	0.66	0.29
**P08228**	Superoxide dismutase [Cu-Zn] OS=Mus musculus	7.33	3.50	1.72	0.48	0.49	0.24
**P56959**	RNA-binding protein FUS	7.33	3.50	5.17	0.48	1.48	0.71
**Q6NZJ6**	Isoform 2 of Eukaryotic translation initiation factor 4 gamma 1	7.33	3.50	3.44	0.48	0.98	0.47
**Q921F2**	TAR DNA-binding protein 43	7.33	3.50	6.89	0.48	1.97	0.94
**Q9EQU5**	Isoform 2 of Protein SET	7.33	3.50	5.17	0.48	1.48	0.71
**Q6ZQ38**	Cullin-associated NEDD8-dissociated protein 1	17.59	10.49	5.17	0.60	0.49	0.29
**P17427**	AP-2 complex subunit alpha-2	2.93	1.75	5.17	0.60	2.95	1.76
**P52293**	Importin subunit alpha-1	2.93	1.75	6.89	0.60	3.94	2.35
**P61161**	Actin-related protein 2	10.26	7.00	3.44	0.68	0.49	0.34
**Q60865**	Caprin-1	8.79	7.00	1.72	0.80	0.25	0.20
**P34022**	Ran-specific GTPase-activating protein	4.40	3.50	8.61	0.80	2.46	1.96
**O35344**	Importin subunit alpha-4	5.86	5.25	1.72	0.89	0.33	0.29
**Q9CQE8**	RNA transcription, translation and transport factor protein	5.86	5.25	1.72	0.89	0.33	0.29
**Q9D6Z1**	Nucleolar protein 56	5.86	5.25	1.72	0.89	0.33	0.29
**P46935**	E3 ubiquitin-protein ligase NEDD4	7.33	7.00	3.44	0.95	0.49	0.47
**Q9ERK4**	Exportin-2	8.79	10.49	5.17	1.19	0.49	0.59
**P23116**	Eukaryotic translation initiation factor 3 subunit A	5.86	7.00	1.72	1.19	0.25	0.29
**P24369**	Peptidyl-prolyl cis-trans isomerase B	5.86	7.00	3.44	1.19	0.49	0.59
**Q9Z1Q5**	Chloride intracellular channel protein 1	5.86	7.00	3.44	1.19	0.49	0.59
**P08003**	Protein disulfide-isomerase A4	4.40	5.25	1.72	1.19	0.33	0.39
**P14733**	Lamin-B1	4.40	5.25	1.72	1.19	0.33	0.39
**P62849**	Isoform 2 of 40S ribosomal protein S24	4.40	5.25	1.72	1.19	0.33	0.39
**P63158**	High mobility group protein B1	4.40	5.25	1.72	1.19	0.33	0.39
**Q64514**	Isoform Short of Tripeptidyl-peptidase 2	4.40	5.25	1.72	1.19	0.33	0.39
**Q99L47**	Hsc70-interacting protein	4.40	5.25	1.72	1.19	0.33	0.39
**O35737**	Heterogeneous nuclear ribonucleoprotein H	7.33	10.49	5.17	1.43	0.49	0.71
**Q60817**	Nascent polypeptide-associated complex subunit alpha	7.33	10.49	5.17	1.43	0.49	0.71
**Q99PL5**	Ribosome-binding protein 1	7.33	10.49	5.17	1.43	0.49	0.71
**Q9R0N0**	Galactokinase	7.33	10.49	5.17	1.43	0.49	0.71
**Q9JMH6**	Isoform 2 of Thioredoxin reductase 1, cytoplasmic	14.66	20.99	10.33	1.43	0.49	0.71
**P11087**	Isoform 2 of Collagen alpha-1(I) chain	4.40	7.00	1.72	1.59	0.25	0.39
**Q9D819**	Inorganic pyrophosphatase	4.40	7.00	3.44	1.59	0.49	0.78
**P37889**	Isoform 2 of Fibulin-2	5.86	10.49	5.17	1.79	0.49	0.88
**Q3THK7**	GMP synthase [glutamine-hydrolyzing]	2.93	5.25	1.72	1.79	0.33	0.59
**Q9QZE7**	Translin-associated protein X	2.93	5.25	1.72	1.79	0.33	0.59
**P47757**	Isoform 2 of F-actin-capping protein subunit beta	10.26	19.24	8.61	1.88	0.45	0.84
**O09061**	Proteasome subunit beta type-1	4.40	8.74	1.72	1.99	0.20	0.39
**Q60716**	Isoform IIa of Prolyl 4-hydroxylase subunit alpha-2	4.40	8.74	3.44	1.99	0.39	0.78
**Q9Z1Q9**	Valine--tRNA ligase	11.73	24.48	10.33	2.09	0.42	0.88
**Q9CZD3**	Glycine--tRNA ligase	5.86	13.99	6.89	2.39	0.49	1.18
**P62874**	Guanine nucleotide-binding protein G(I)/G(S)/G(T) subunit beta-1	2.93	7.00	3.44	2.39	0.49	1.18
**P10852**	4F2 cell-surface antigen heavy chain	1.47	5.25	1.72	3.58	0.33	1.18
**P56655**	Cytochrome P450 2C38	1.47	5.25	1.72	3.58	0.33	1.18
**Q9WUM4**	Coronin-1C	1.47	5.25	1.72	3.58	0.33	1.18
**Q60715**	Prolyl 4-hydroxylase subunit alpha-1	2.93	13.99	6.89	4.77	0.49	2.35

Table.S2 : Cell Culture and Pharmacological Reagents

Cell Culture and Pharmacological Reagents		Final Concentration
IMDM	GIBCO	-
FCS	Atlanta Biologicals	10% v/v
Penicillin/streptomycin	GIBCO	1% v/v

Table.S3 : Antibodies used and their final concentrations for western blots

Antibodies		Final Concentration
Akt (4685)	Cell Signaling	1/2000
p-Akt Ser473 (4058L)	Cell Signaling	1/1000
LaminA/C (4C11)	Cell Signaling	1/1000
p-FAK Tyr397 (328 3)	Cell Signaling	1/1000
FAK (sc-558)	Santa Cruz Biotechnology	1/500
Lamin B1 (OAEB03011)	Aviva Systems Biology	1/1000
Vinculin (4650T)	Cell Signaling	1/500
Sun-1 (HPA008346)	Sigma Aldrich	1/1000
Sun-2 (ab87036)	Abcam	1/1000
GAPDH (5174S)	Cell Signaling	1/1000
YAP (14074S)	Cell Signaling	1/1000

Table.S4 : siRNA sequences

siRNA Sequences -Stealth Select siRNAs (Invitrogen)	
siRNA SUN-1 Control	5′- GAAATCGAAGTACCTCGAGTGATAT -3′
siRNA SUN-1	5′- GAAAGGCTATGAATCCAGAGCTTAT-3′
siRNA SUN-2 Control	5′-CACCAGAGGCTAGAACTCTTACTCA-3′
siRNA SUN-2	5′-CACCAGAGGCTAGAACTCTTACTCA-3′

Table.S5: PCR Primers used

PCR primers	
Sun-1 -Forward	5′-CATGAAGTGCGTCTCTCCAA-3′
Sun-1 -Reverse	5′- CCTGCTTTCAGCTTGGTTTC-3′
Sun-2 -Forward	5′-CAGCAACATGAAGCACCTGT-3′
Sun-2 -Reverse	5′-GCTGCCGATGTAGGACTCTC-3′
Lmna -Forward	5′- TGAGTACAACCTGCGCTCAC -3
Lmna -Reverse	5′- TGTGACACTGGAGGCAGAAG
Nesprin1G -Forward	5′-AAGCAACTCAAGGCTTTCCA-3′
Nesprin1G -Reverse	5′-GTCTGAGCTCGTCCAGTTCC-3′
Nesprin2G -Forward	5′- ACCTCGCCAACACATACCTC-3′,
Nesprin2G -Reverse	5′- TGCTTCTTGCTGCTTCTTCA-3′
18S –Forward	5′-GAACGTCTGCCCTATCAACT-3′
18S -Reverse	5′-CCAAGATCCAACTACGAGCT-3

Table.S6: Immunostaining antibodies and reagents and their final concentrations

Immunostaining antibodies and Reagents		Final Concentration
Hoechst 33342	Invitrogen	1 μg/mL
Alexa Fluor 488 Phalloidin	Life Technologies	0.1μM

Figure S3 Unprocessed Blots

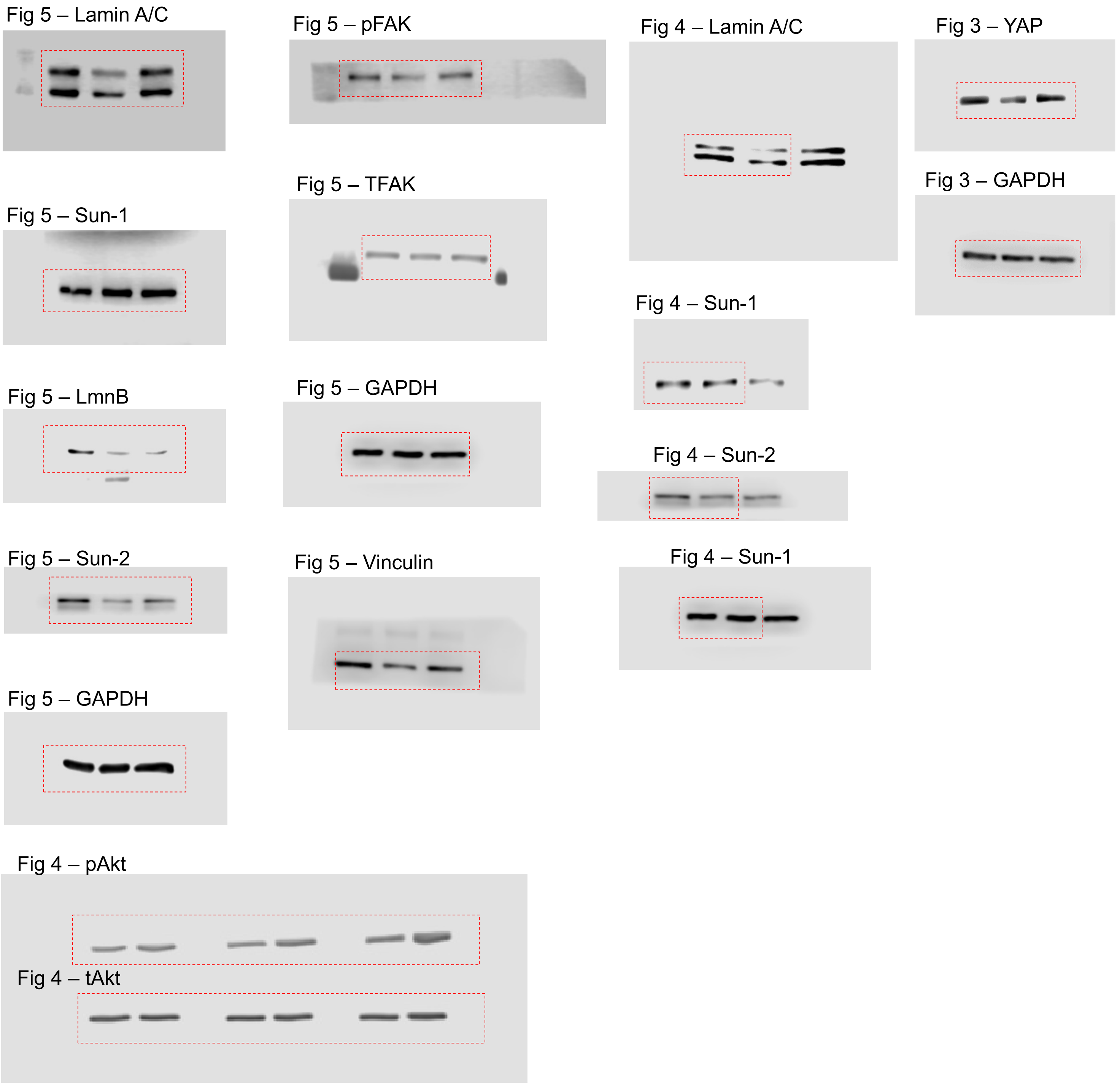

Figure S3. Unprocessed blots as obtained by LiCor C-DiGit blot scanner.
