# Supplementary material for: Recovery of stem cell proliferation by low intensity vibration under simulated microgravity requires LINC complex
Source: NPJ Microgravity. 2019 May 15;5:11. doi: 10.1038/s41526-019-0072-5 (PMC6520402; doi:10.1038/s41526-019-0072-5)
Supplement: Supplementary file 2 — Reporting Summary [file 41526_2019_72_MOESM2_ESM.pdf]

nature research

Corresponding author(s): Gunes Uzer

Last updated by author(s): Jan 28, 2019

Reporting Summary

Nature Research wishes to improve the reproducibility of the work that we publish. This form provides structure for consistency and transparency in reporting. For further information on Nature Research policies, see Authors & Referees and the Editorial Policy Checklist.

Statistics

For all statistical analyses, confirm that the following items are present in the figure legend, table legend, main text, or Methods section.

n/a	Confirmed	
☐	☒	The exact sample size (*n*) for each experimental group/condition, given as a discrete number and unit of measurement
☐	☒	A statement on whether measurements were taken from distinct samples or whether the same sample was measured repeatedly
☐	☒	The statistical test(s) used AND whether they are one- or two-sided<br>*Only common tests should be described solely by name; describe more complex techniques in the Methods section.*
☒	☐	A description of all covariates tested
☐	☒	A description of any assumptions or corrections, such as tests of normality and adjustment for multiple comparisons
☐	☒	A full description of the statistical parameters including central tendency (e.g. means) or other basic estimates (e.g. regression coefficient) AND variation (e.g. standard deviation) or associated estimates of uncertainty (e.g. confidence intervals)
☐	☒	For null hypothesis testing, the test statistic (e.g. *F*, *t*, *r*) with confidence intervals, effect sizes, degrees of freedom and *P* value noted<br>*Give P values as exact values whenever suitable.*
☒	☐	For Bayesian analysis, information on the choice of priors and Markov chain Monte Carlo settings
☒	☐	For hierarchical and complex designs, identification of the appropriate level for tests and full reporting of outcomes
☒	☐	Estimates of effect sizes (e.g. Cohen's *d*, Pearson's *r*), indicating how they were calculated

Our web collection on statistics for biologists contains articles on many of the points above.

Software and code

Policy information about availability of computer code

Data collection	Image Studio, Ver 5.2, Roche Lightcycler 1.0, Velos Pro Dual-Pressure Linear Ion Trap mass spectrometer (Thermo Fisher Scientific)
Data analysis	Excel, NIH imageJ software (https://imagej.nih.gov/ij/)

For manuscripts utilizing custom algorithms or software that are central to the research but not yet described in published literature, software must be made available to editors/reviewers. We strongly encourage code deposition in a community repository (e.g. GitHub). See the Nature Research guidelines for submitting code & software for further information.

Data

Policy information about availability of data

All manuscripts must include a data availability statement. This statement should provide the following information, where applicable:

- Accession codes, unique identifiers, or web links for publicly available datasets
- A list of figures that have associated raw data
- A description of any restrictions on data availability

The datasets generated during and/or analysed during the current study are available from the corresponding author on reasonable request.

Field-specific reporting

Please select the one below that is the best fit for your research. If you are not sure, read the appropriate sections before making your selection.

☒ Life sciences ☐ Behavioural & social sciences ☐ Ecological, evolutionary & environmental sciences

For a reference copy of the document with all sections, see nature.com/documents/nr-reporting-summary-flat.pdf

Life sciences study design

All studies must disclose on these points even when the disclosure is negative.

Sample size	For LIV samples, Sample sizes were determined via power calculators using estimated differences and data variability based on our previous experiments we utilized our previously published data on cell proliferation of health cells to reach 80% power. We did not perform any predetermined methods to assign sample before starting the sMG experiments but used our preliminary data to determine required sample size via power calculators using estimated differences and data variability based on our preliminary testing.
Data exclusions	No data exclusion was performed.
Replication	All data had three technical replicates and experiments independently repeated at least three times.
Randomization	Samples were assigned randomly to each group for each experiment during the start of the experiment.
Blinding	When samples were assigned randomly to each group for each experiment during the start, researcher became aware of the group assignments as they had to treat them for sMG and or LIV. As same researchers did the data analysis they were not blinded. Mass spec analysis was blinded

Reporting for specific materials, systems and methods

We require information from authors about some types of materials, experimental systems and methods used in many studies. Here, indicate whether each material, system or method listed is relevant to your study. If you are not sure if a list item applies to your research, read the appropriate section before selecting a response.

Materials & experimental systems

n/a	Involved in the study	
☐	☒	Antibodies
☐	☒	Eukaryotic cell lines
☒	☐	Palaeontology
☒	☐	Animals and other organisms
☒	☐	Human research participants
☒	☐	Clinical data

Methods

n/a	Involved in the study	
☒	☐	ChIP-seq
☒	☐	Flow cytometry
☒	☐	MRI-based neuroimaging

Antibodies

Antibodies used

Akt (4685) Cell Signaling 1/2000
p-Akt Ser473 (4058L) Cell Signaling 1/1000
LaminA/C (4C11) Cell Signaling 1/1000
p-FAK Tyr397 (328 3) Cell Signaling 1/1000
FAK (sc-558) Santa Cruz Biotechnology 1/500
Lamin B1 (OAEB03011) Aviva Systems Biology 1/1000
Vinculin (4650T) Cell Signaling 1/500
Sun-1 (HPA008346) Sigma Aldrich 1/1000
Sun-2 (ab87036) Abcam 1/1000
GAPDH (5174S) Cell Signaling 1/1000
YAP (14074S) Cell Signaling 1/1000

All antibodies are used for western blotting.

Validation

All antibodies used were validated by the companies as sated below.

Akt (4685) Cell Signaling - Akt (pan) (11E7) Rabbit mAb detects endogenous levels of total Akt protein. This antibody does not cross-react with other related proteins. Species Reactivity: Human, Mouse, Rat, Monkey.
p-Akt Ser473 (4058L) Cell Signaling - Phospho-Akt (Ser473) (193H12) Rabbit mAb detects endogenous levels of Akt only when phosphorylated at Ser473. Species Reactivity: Human, Mouse, Rat
LaminA/C (4C11) Cell Signaling - Lamin A/C (4C11) Mouse mAb detects endogenous levels of lamin A and lamin C proteins. It also reacts with the larger fragments of lamin A (50 kDa) and lamin C (41 kDa) produced by caspase cleavage during apoptosis. This antibody does not cross-react with lamins B1 and B2.Species Reactivity: Human, Mouse, Rat, Monkey
p-FAK Tyr397 (328 3) Cell Signaling - Phospho-FAK (Tyr397) Antibody detects endogenous levels of FAK only when phosphorylated at Tyr397. This antibody may cross-reacts with other tyrosine-phosphorylated RTKs. Species Reactivity: Human, Mouse, Rat, Hamster, Pig
FAK (sc-558) Santa Cruz Biotechnology 1/500- FAK (C-20) is recommended fordetection of FAKp125 and FRNKp4 of mouse,rat , human,chicken,Xenopuslaevisandzebrafish origin by Western Blotting.
Vinculin (4650T) Cell Signaling 1/500- Vinculin Antibody detects endogenous levels of total vinculin protein. This antibody also

reacts with metavinculin, a 145 kDa splice variant of vinculin.Species Reactivity: Human, Mouse, Rat, Monkey, Dog
Sun-1 (HPA008346) Sigma Aldrich 1/1000 - Anti-SUN1 antibody produced in rabbit, a Prestige Antibody, is developed and validated by the Human Protein Atlas (HPA) project (www.proteinatlas.org). Each antibody is tested by immunohistochemistry against hundreds of normal and disease tissues. These images can be viewed on the Human Protein Atlas (HPA) site by clicking on the Image Gallery link. The antibodies are also tested using western blotting. Immunogen sequence also predicted to interact with Mouse and we have tested this in our cells.
Sun-2 (ab87036) Abcam - Reacts with N terminal amino acids 1-18 of Mouse SUN2, tested by abcam.
GAPDH (5174S) Cell Signaling 1/1000 - DH (D16H11) XP® Rabbit mAb detects endogenous levels of total GAPDH protein.Species Reactivity:Human, Mouse, Rat, Monkey Species predicted to react based on 100% sequence homology: Pig
YAP (14074S) Cell Signaling 1/1000- YAP (D8H1X) XP® Rabbit mAb recognizes endogenous levels of total YAP protein. Species Reactivity: Human, Mouse, Rat, Hamster, Monkey Species predicted to react based on 100% sequence homology: Bovine, Guinea Pig, Horse

Eukaryotic cell lines

Policy information about cell lines

Cell line source(s)	Primary MSCs
Authentication	Cells were checked for differentiation potential.
Mycoplasma contamination	No mycoplasma contamination was detected.
Commonly misidentified lines (See ICLAC register)	N/A
